# Application of collapsing methods for continuous traits to the Genetic Analysis Workshop 17 exome sequence data

**DOI:** 10.1186/1753-6561-5-S9-S121

**Published:** 2011-11-29

**Authors:** Yun Ju Sung, Treva K Rice, Dabeeru C Rao

**Affiliations:** 1 Division of Biostatistics, Washington University School of Medicine, 660 S. Euclid Ave., St. Louis, MO 63110, USA

## Abstract

Genetic Analysis Workshop 17 used real sequence data from the 1000 Genomes Project and simulated phenotypes influenced by a large number of rare variants. Our aim is to evaluate the performance of various collapsing methods that were developed for analysis of multiple rare variants. We apply collapsing methods to continuous phenotypes Q1 and Q2 for all 200 replicates of the unrelated individuals data. Within each gene, we collapse (1) all SNPs, (2) all SNPs with minor allele frequency (MAF) < 0.05, and (3) nonsynonymous SNPs with MAF < 0.05. We consider two tests when collapsing variants: using the proportion of variants and using the presence/absence of any variant. We also compare our results to a single-marker analysis using PLINK. For phenotype Q1, the proportion test for collapsing rare nonsynonymous SNPs often performed the best. Two genes (*FLT1* and *KDR*) had statistically significant results. A single-marker analysis using PLINK also provided statistically significant results for some SNPs within these two genes. For phenotype Q2, collapsing rare nonsynonymous SNPs performed the best, with almost no difference between proportion and presence tests. However, neither collapsing methods nor a single-marker analysis provided statistically significant results at the true genes for Q2. We also found that a large number of noncausal genes had high correlations with causal genes for Q1 and Q2, which may account for inflated false positives.

## Background

Statistical power to identify rare variants is limited because of the small number of observations for any given variant. As Dering et al. [1] summarized, several collapsing methods for identifying rare variants have focused on testing the combined effect of multiple rare variants. For continuous traits, we apply the two tests from Morris and Zeggini [2] that accumulate minor alleles within the same functional unit. The first test uses the proportion of rare variants at which an individual carries a minor allele, whereas the second test uses the presence or absence of a minor allele at any rare variant within an individual.

The Genetic Analysis Workshop 17 (GAW17) simulation uses real sequence data from the 1000 Genomes Project and simulated phenotypes influenced by a large number of rare variants [3]. Our main goal in this paper is to investigate the performance of various collapsing methods on the GAW17 exome sequence data set. Also, we compare the gain in power of collapsing methods relative to single-marker analysis using PLINK [4]. Analyses were performed without knowledge of the underlying simulation model. However, we use the GAW17 answers in presenting the results.

## Methods

### Data and covariate adjustment

We use all 200 replicates of phenotypes Q1 and Q2 for the unrelated individuals data set provided by GAW17. The GAW17 exome sequence data include 24,487 single-nucleotide polymorphisms (SNPs) in 3,205 genes. Out of 3,205 genes, 1,218 genes have only a single SNP, for which a collapsing method is identical to a single-SNP analysis. We exclude 15 genes because all SNPs in these genes are classified neither synonymous nor non-synonymous. We apply collapsing methods to the remaining 1,972 genes.

To adjust for possible covariates, we run a single-marker analysis using PLINK for the first replicate with three possible covariates (Age, Sex, and Smoking status); this analysis corresponds to a multiple linear regression that regresses continuous phenotypes Q1 and Q2 on the additive coding of SNP effect and three covariates. Age and Smoking status are significant covariates for Q1. The average −log(*P*) across all 24,487 SNPs is 18.6 for Age and 9.9 for Smoking status. However, there is no significant covariate for Q2. These findings are consistent with the GAW17 simulation answers. Significant covariates are included for the remaining analysis for both single-marker and collapsing methods for all 200 replicates.

### Collapsing and single-marker methods

We run collapsing methods using R for the 1,972 genes that include more than 1 SNP and a single-marker analysis using PLINK for 24,487 SNPs. Within a gene, we consider three different collapsing methods: (1) collapsing all variants, (2) collapsing variants with minor allele frequency (MAF) < 0.05, and (3) collapsing nonsynonymous SNPs with MAF < 0.05. For each collapsing method, we apply both the proportion and presence/absence tests of Morris and Zeggini [2].

To evaluate the performance of various collapsing methods, we compute true-positive (power) and false-positive (type I error) rates for each level *α* and plot the receiver operating characteristic (ROC) curve using these true- and false-positive rates across *α*. The true-positive rate is computed by averaging the proportion of replicates with *P* ≤ *α* over 200 replicates across true genes; the false-positive rate is computed similarly.

In addition to power and type I error rates, we also use −log(*P*) values to compare collapsing methods to single-marker analysis. In a single-marker analysis, the Manhattan plot that shows −log(*P*) across the genome is commonly used to visually assess statistical significance of association. To summarize results from all 200 replicates, we use −log(*P*) averaged across 200 replicates. Note that the average of −log(*P*) can linearly correspond to Fisher’s method of combining *p*-values across studies in a meta-analysis. From single-marker analysis, we use the highest value among the average −log(*P*) values across 200 replicates of all SNPs within each gene.

## Results

### Collapsing methods: proportion vs. presence tests

**Figure 1:**
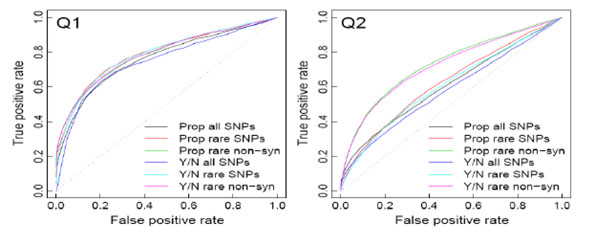
**ROC curves of collapsing methods for Q1 and Q2**. The *y*-axis shows power (true-positive rate) and the *x*-axis shows type I error rate (false-positive rate) for significance level between 0 and 1. Prop is the proportion test; Y/N is the presence test.

Overall performance of collapsing methods is presented as ROC curves (Figure 1), showing true- and false-positive rates for phenotypes Q1 and Q2. Collapsing rare nonsynonymous SNPs provided the best results for both phenotypes, providing the highest power for every type I error rate. The advantage of collapsing nonsynonymous SNPs was more pronounced for Q2 than for Q1. Results of proportion and presence/absence tests were similar, and their ROC curves were hardly distinguishable. As shown in Table 1, collapsing methods performed better for phenotype Q1 than for Q2.

**Table 1:** Averaged −log(*P*) across 200 replicates from collapsing methods for Q1 and Q2 at the true causal genes

Gene	All SNPs			Rare SNPs			Rare nonsynonymous SNPs			Best single
		
	*N* (true)	Prop	Y/N	*N*	Prop	Y/N	*N*	Prop	Y/N	
Q1										
*ARNT*	18 (5)	0.45	0.40	17	1.88	1.88	9	2.24	2.50	3.94
*ELAVL4*	10 (2)	**2.47**	2.38	8	0.85	0.85	4	0.76	0.76	2.28
*FLT1*	35 (11)	6.61	2.59	32	9.70	6.84	19	**17.81**	15.04	24.26
*FLT4*	10 (2)	2.70	2.98	10	2.70	**2.98**	5	1.44	1.50	1.77
*HIF1A*	8 (4)	3.03	3.31	8	3.03	3.31	6	3.24	**3.48**	3.39
*HIF3A*	21 (3)	**0.62**	0.37	17	0.34	0.36	8	0.55	0.58	1.02
*KDR*	16 (10)	8.78	3.84	15	8.24	8.18	10	**8.77**	8.10	5.63
*VEGFA*	6 (1)	1.47	1.64	6	1.47	**1.64**	2	0.87	0.87	1.21
Q2										
*BCHE*	29 (13)	0.71	0.57	28	1.22	1.43	25	1.32	1.59	1.58
*INSIG1*	5 (3)	0.53	0.50	5	**0.53**	0.50	4	0.39	0.39	0.57
*LPL*	20 (3)	0.37	0.37	17	0.64	0.63	8	**1.38**	1.27	1.88
*PDGFD*	11 (4)	0.44	0.50	9	0.72	0.94	7	**1.66**	1.66	1.81
*PLAT*	29 (8)	0.41	0.37	27	0.42	0.46	11	0.86	**0.89**	0.95
*RARB*	11 (2)	0.66	0.62	11	0.66	0.62	3	1.26	**1.26**	1.22
*SIRT1*	24 (9)	**1.89**	1.25	23	1.60	1.04	14	1.34	1.20	2.03
*SREBF1*	24 (10)	0.52	0.89	23	**0.91**	0.87	18	0.80	0.76	1.12
*VLDLR*	27 (8)	0.41	0.40	24	0.65	0.61	15	**1.23**	1.22	0.83
*VNN1*	7 (2)	**3.24**	2.90	6	0.37	0.46	2	0.96	0.96	3.42
*VNN3*	15 (7)	**1.29**	0.80	12	0.94	0.91	9	1.03	1.01	2.08
*VWF*	8 (2)	0.89	0.54	8	**0.89**	0.54	4	0.75	0.59	0.90

Boldface indicates the best result among the collapsing methods for each gene. Rare SNPs are defined as SNPs with MAF < 0.05. Prop is the proportion test; Y/N is the presence test. “Best Single” is the best signal from a single-marker analysis among all SNPs within each gene.

A more detailed performance of the collapsing methods at the true causal genes is presented in Table 1 for phenotypes Q1 and Q2. The Bonferroni-corrected threshold for 1,972 genes is −log(0.05/1,972) = 4.59. For Q1, in the first replicate, the highest signal was 21.39 at the *FLT1* gene from the proportion test for collapsing rare nonsynonymous SNPs. In 200 replicates, all tests except the presence test for collapsing all variants gave statistically significant results (Table 1). However, the proportion test for collapsing rare nonsynonymous SNPs gave the most significant results, with −log(*P*) ranging from 8.29 to 25.24 (mean 17.76) across 200 replicates. This was particularly interesting because the causal SNP C13S523 had MAF = 0.07 and was excluded from collapsing. The next highest signal was 10.38 at the *KDR* gene, also from the proportion test for collapsing rare nonsynonymous SNPs. In contrast to the *FLT1* gene, all tests except the presence test for collapsing all SNPs gave similar results for the *KDR* gene. We suspect that this happened because the *KDR* gene has the causal SNP C4S1878 with MAF = 0.16 and also because noise from noncausal SNPs for the *KDR* gene was relatively lower than that for the *FLT1* gene. No other genes were statistically significant.

For phenotype Q2, no true gene was statistically significant for the first replicate and also across 200 replicates on average (Table 1). The highest signal was 3.24 at the *VNN1* gene from the proportion test for collapsing all SNPs, ranging from 0.38 to 7.41 across 200 replicates. The signal was dramatically reduced when collapsing only rare SNPs. We suspect that this happened because the *VNN1* gene had only two causal SNPs and one of them had MAF = 0.17. Hence there was less gain in power for the collapsing methods relative to a single-marker analysis.

### Single-marker analysis using PLINK

**Figure 2:**
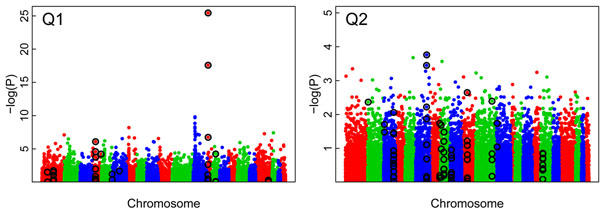
**Manhattan plots for Q1 and Q2 using the first replicate from the single-marker analysis using PLINK.** The *x*-axis represents chromosomes 1 through 22, and the *y*-axis is the −log(*P*) value for the association test. The black circles indicate the true causal SNPs.

Figure 2 presents results from genome-wide association studies for phenotypes Q1 and Q2 using PLINK for the first replicate. The Bonferroni-corrected threshold for 24,487 SNPs is −log(0.05/24,487) = 5.69. For Q1, there were 34 statistically significant SNPs, among which 3 were causal: C13S523 (MAF = 0.06) and C13S522 (MAF = 0.03) in the *FLT1* gene and C4S1884 (MAF = 0.02) in the *KDR* gene. The most common SNP among the causal SNPs was C4S1878 (MAF = 0.16), also in the *KDR* gene, but it failed to reach significance (−log(*P*) = 4.44). Across all 200 replicates, the most significant SNPs among causal SNPs were C13S523, C13S522, C13S524, C4S1877, and C4S1889; their mean −log(*P*) values were 24.26, 13.84, 6.55, 5.63, and 5.63, respectively.

For Q2, no SNP was statistically significant in the first replicate, as shown in Figure 2. The highest signal was 3.76, which occurred at the causal SNP C6S5449 (MAF = 0.01) in the *VNN3* gene; across all 200 replicates, its −log(*P*) value ranged from 0.00 to 9.74, with a mean of 2.08. The next highest signal among causal SNPs was 3.45, which occurred at the most common causal SNP, C6S5380 (MAF = 0.17), in the *VNN1* gene; across 200 replicates, its −log(*P*) value ranged from 0.37 to 8.09, with a mean of 3.42.

### Private mutations and inflated false positives

Many GAW17 investigators have observed inflated false positives for various collapsing methods. We found in the GAW17 data that 9,433 (out of 24,487) SNPs were private mutations, having only a single copy in the entire group of 697 subjects. These private mutations occurred in 685 subjects. Each subject had an average of 14 private mutations. The extreme cases were subject NA19327 with 155 private mutations and subject NA19319 with 77 private mutations. Because genotypes for private mutations that occurred in the same subject were identical, their correlations were 1; hence a causal variant that is a private mutation has correlation 1 with the other private mutations in the same subject. We found that 386 private mutations were perfectly correlated with one of 23 causal private mutations for Q1, and 738 private mutations were perfectly correlated with one of 37 causal private mutations for Q2. These mutations were in 266 and 504 other noncausal genes for Q1 and Q2, respectively. Because a large number of these noncausal genes were highly correlated with causal genes, they are suspected of inflating type I errors.

## Discussion and conclusions

We applied several collapsing methods to the GAW17 exome sequence data. For phenotype Q1, the proportion test for collapsing rare nonsynonymous SNPs often performed the best. Two causal genes, *FLT1* and *KDR*, had statistically significant results. A single-marker analysis using PLINK also provided statistically significant results for some SNPs within these two genes. For phenotype Q2, collapsing rare nonsynonymous SNPs provided much better performance than other collapsing methods (as shown by the ROC curves). However, neither collapsing methods nor single-marker analysis provided statistically significant results at the true causal genes.

We observed several important findings from applying collapsing methods to the GAW17 exome sequence data. First, collapsing methods did not seem to provide additional power over the single-marker analysis. Second, various collapsing methods appeared to be similar. Third, their performance for phenotypes Q1 and Q2 was a bit disappointing. Many GAW17 investigators have observed inflated type I error rates for these collapsing methods. Luedtke et al. [5] identified 695 spuriously associated genes that showed consistent association with the discrete phenotype Affected, which is consistent with our findings about noncausal genes having high correlations with causal genes.

GAW17 provided an opportunity to evaluate collapsing methods using sequence data. We would like to emphasize two observations. First, 38% of genes (1,218 out of 3,205) have only a single SNP, for which a collapsing method is identical to a single-SNP analysis. Because the GAW17 data were based on the real targeted exome sequence data from the pilot3 study of the 1000 Genomes Project, other exome sequence data are likely to have a similar feature. Here, we simply excluded these genes from consideration. However, collapsing variants across multiple genes might be a better approach. This approach might also work for other sequence data that include a large number of SNPs that do not belong to any gene. Second, 38.5% of SNPs (9,433 out of 24,487) were private mutations, having only a single copy in the entire set of 697 subjects. Some of these may be sequence errors. These mutations create correlations across multiple chromosomes. Even though these rare variants can be causal, as simulated in the GAW17 data, it is not possible to identify which variants are causal among multiple private mutations in a single subject. The 1000 Genomes Project recently released sequence data that contain more than 16 million SNPs for 629 subjects. Their sequence data are also likely to contain a large number of private mutations, which would raise similar issues.

## Competing interests

The authors declare that they have no competing interests.

## Authors’ contributions

YJS conceived of the study, carried out the analysis, and drafted the manuscript. TKR conceived of the covariate adjustment scheme and summarized the results. DCR participated in the design of the study and helped to draft the manuscript. All authors read and approved the final manuscript.
